# Case report: upper arm metastasis of an oral squamous cell carcinoma

**DOI:** 10.1186/s12903-015-0007-9

**Published:** 2015-02-15

**Authors:** Matthias Christian Wurm, Emeka Nkenke, Friedrich-Wilhelm Neukam, Tobias Möst, Rainer Lutz, Falk Wehrhan, Kathrin Brunner, Konstantinos Theodorou Mitsimponas, Philipp Schlechtweg, Cornelius von Wilmowsky

**Affiliations:** Department of Oral and Maxillofacial Surgery, University of Erlangen-Nürnberg, Glückstrasse 11, 91054 Erlangen, Germany; Department of Oral and Maxillofacial Plastic Surgery, Martin-Luther-University Halle-Wittenberg, Halle, Germany; Department of Pathology, University of Erlangen-Nürnberg, Erlangen, Germany; Department of Radiology, University of Erlangen-Nürnberg, Erlangen, Germany

**Keywords:** Mouth neoplasms, Neoplasm metastasis, Upper arm metastasis, Soft-tissue metastasis, Metastatic OSCC

## Abstract

**Background:**

The Oral Squamous Cell Carcinoma (OSCC) frequently metastasizes lymphogenously. Haematogenous dissemination is less common. This report describes a rare case of a metastatic OSCC of the floor of the mouth to the patients’ left upper arm. To our knowledge this is the first of such case described in the literature.

**Case presentation:**

Twelve months after R0 tumor resection surgery, including microvascular reconstruction of the lower jaw followed by adjuvant radiotherapy, the patient was admitted for osteosynthesis plates removal. During clinical examination a tumor located at his left upper arm was detected. According to the patient the tumor has demonstrated rapid growth. Macroscopic appearance and conventional imaging led to the differential diagnosis of an abscess. MR-imaging could not differentiate between a tumor of soft tissue origin and a metastasis. A biopsy was taken and the pathological examination confirmed the diagnosis of an OSCC metastasis. The postoperative interdisciplinary tumor board recommended radiation therapy.

**Conclusion:**

Due to the fact that patients with regional lymph node metastases have a higher probability to develop distant metastasis a more detailed screening might be considered – especially when hemangiosis carcinomatosa was histologically or macroscopically found.

## Background

The incidence of the Oral Squamous Cell Carcinoma (OSCC) remains high. The five-year survival is about 55% in Germany [1]. The pattern of metastatic dissemination primarily involves the cervical lymph nodes, followed by distant lymph nodes and hematogenous distant metastasis, mainly of the lungs [2,3]. Nevertheless – to our knowledge - an upper arm soft-tissue metastasis of an OSCC of the floor of the mouth has never been described in the literature so far. Here we present the first case of a soft-tissue metastasis of an OSCC in the upper arm region with simultaneous erosion of the humeral bone; given the fact that this diagnosis has been unprecedented, a wide spectrum of clinical entities were applied during the process of the differential diagnoses until the diagnosis was confirmed with a biopsy.

## Case presentation

**Figure 1:**
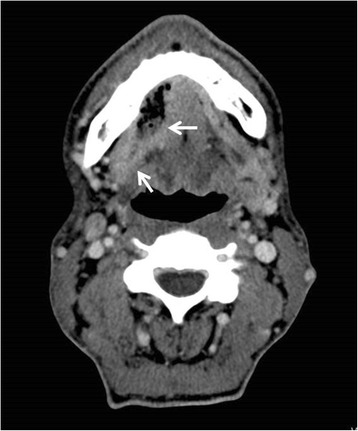
**The computed tomography illustrates a tumor on the right floor of the mouth (arrows).** Radiographically there is the suspicion of an erosion of the cortical bone of the lower jaw.

**Figure 2:**
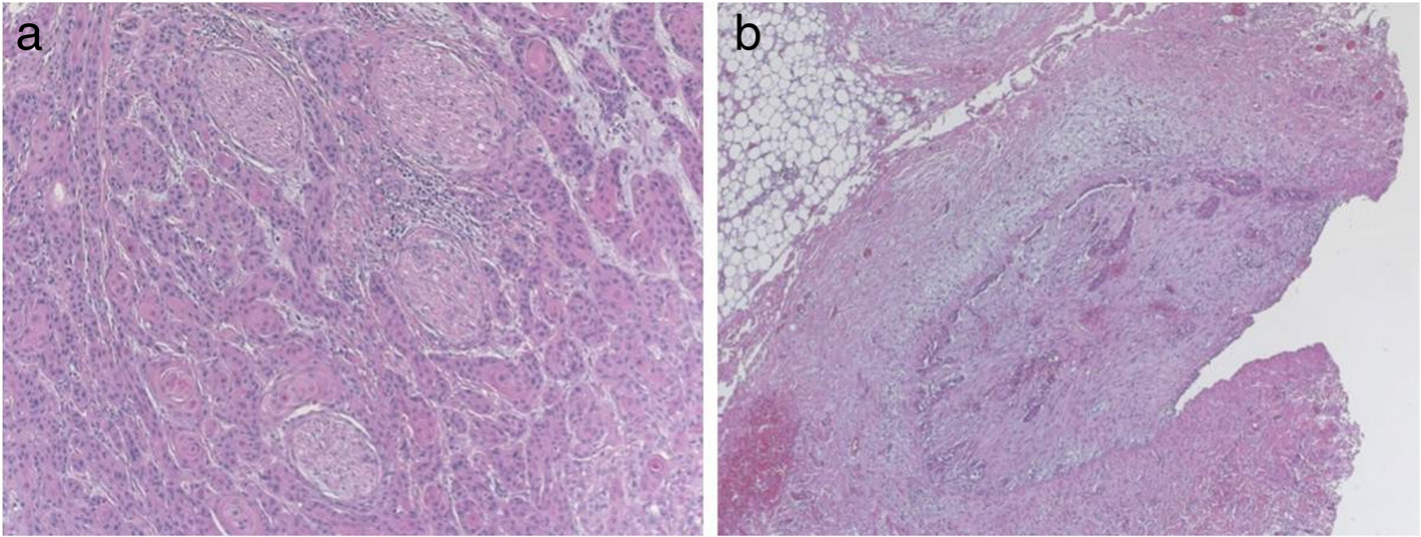
**Microscopic findings of the primary tumor: moderately differentiated squamous cell carcinoma with perineural infiltration (a).** The microscopic findings of a macroscopically visible nodule in the neck dissection specimen (Level Ib, right side) showed a hemangiosis carcinomatosa (V2) **(b)**.

**Figure 3:**
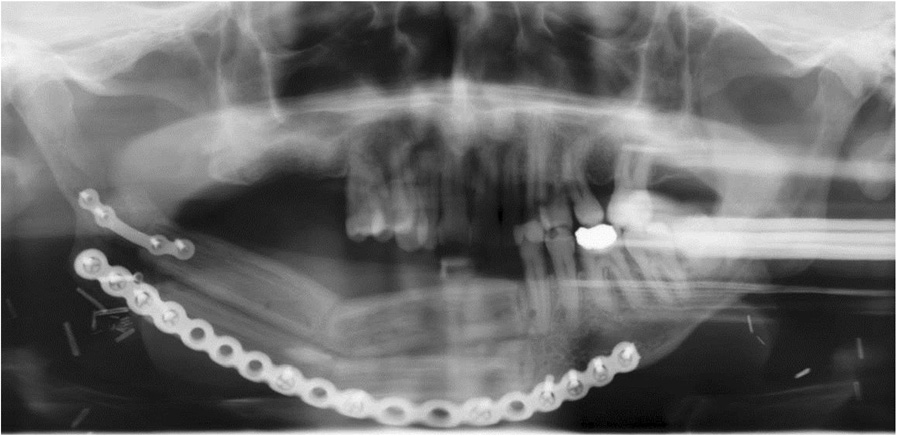
**The panoramic radiograph, taken right before the osteosynthesis plates removal, shows the reconstructed lower jaw with a double-barreled fibula.**

**Figure 4:**
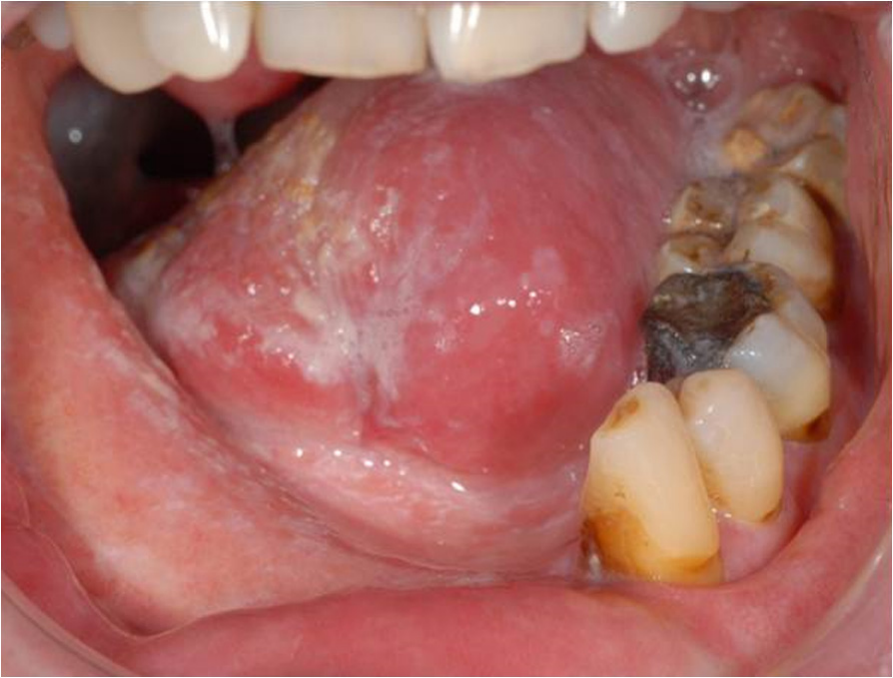
**Showing the patient 12 months after resection of the OSCC of the floor of the mouth (a & b).**

**Figure 5:**
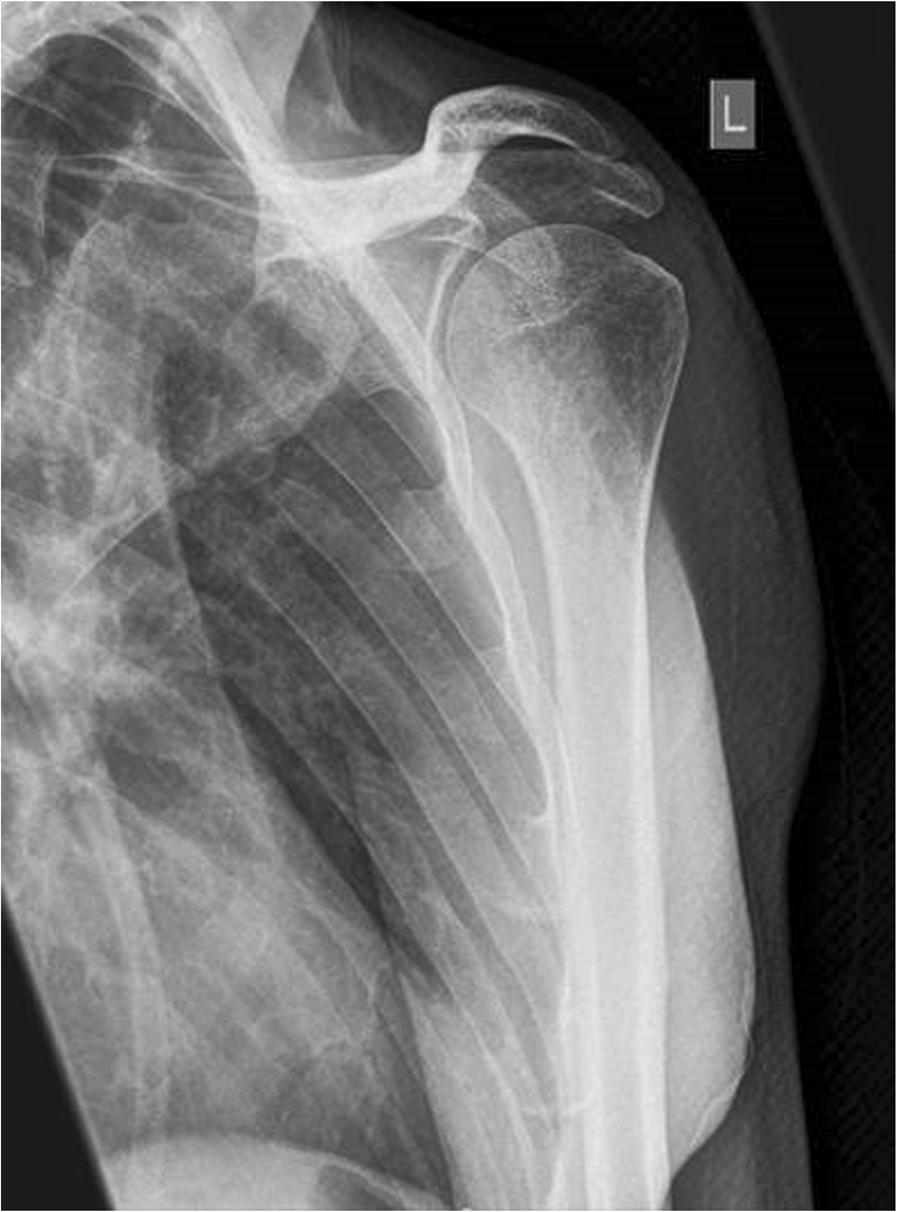
**X-ray of the upper arm shows a swelling of the soft-tissue in projection to the lower margin of the deltoid muscle (arrow).**

**Figure 6:**
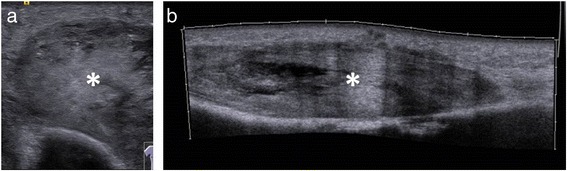
**Sonography of the upper arm shows the tumor (a & b; mark).** A discrimination between abscess and soft-tissue tumor cannot be done.

**Figure 7:**
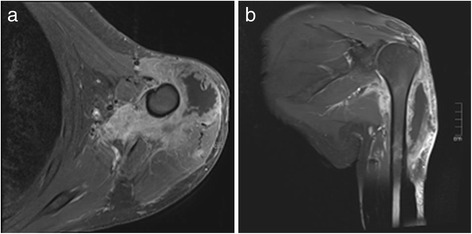
**MR-Imaging of the upper arm in axial (a) and coronal (b) orientation.** Both images (fat saturated T1 weighted Images after contrast administration) show a contrast enhanced soft tissue mass in the proximal upper arm with a circular extent around the proximal humeral bone. Extensive soft tissue involvement suggested malignancy but a definitive diagnosis was not possible. On both images a large necrotic central part without contrast enhancement is visible.

**Figure 8:**
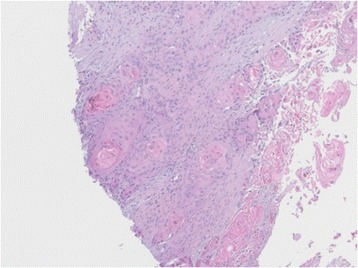
**Microscopic findings of the punch biopsy of the left arm: infiltration by a moderately differentiated squamous cell carcinoma.**

**Figure 9:**
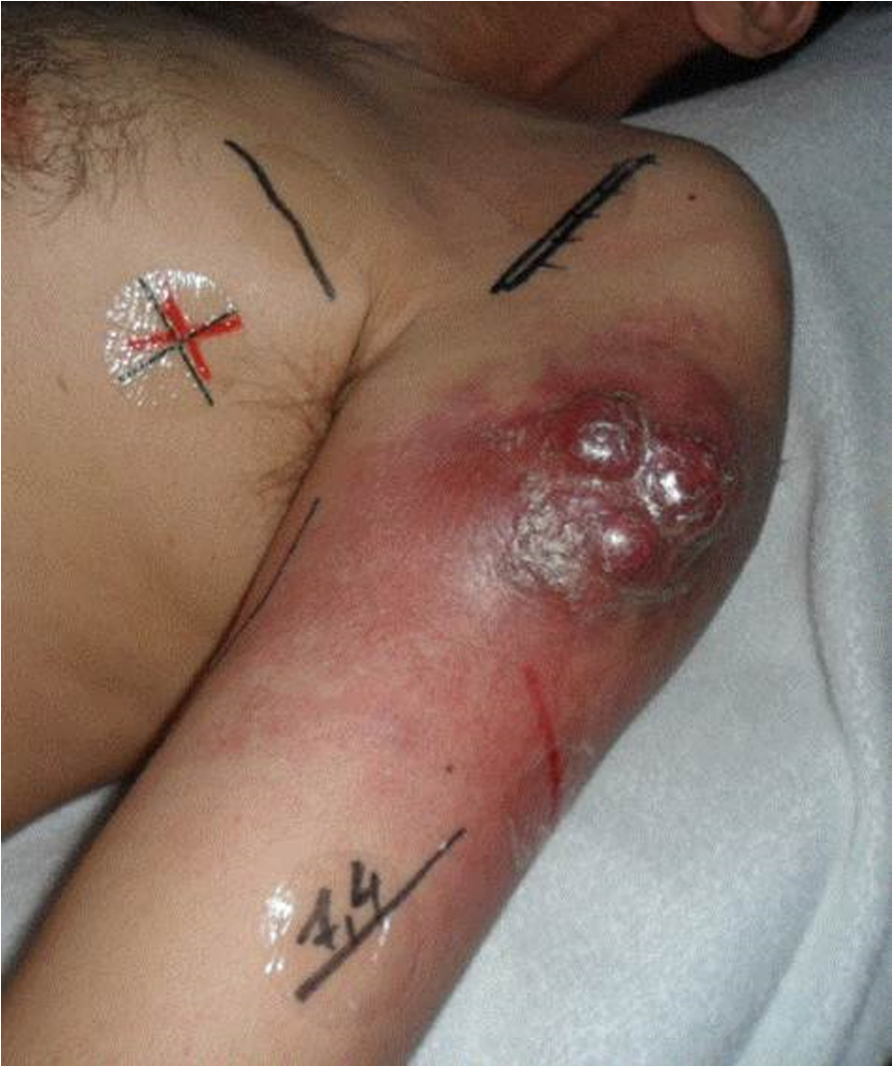
**Upper arm metastasis right before radiation therapy.** Remarkable is the rapid progress of the metastasis after the biopsy was taken.

A written consent for this case report has been obtained by the patient. The patient is a 52-year-old male Caucasian, residing in the south of Germany. There was no specific family history of malignancy. As far as risk factors are concerned the patient has a smoking history adding up to 45 pack years and still smokes about 10 to 15 cigarettes per day. There was no alcohol consumption reported. He was referred by his dentist to our Department in February 2013 with a suspected OSCC of the floor of the mouth. The conducted head and neck computed tomography (CT) strengthened the clinical suspicion of an OSCC of the right floor of the mouth; a tumor that was also infiltrating the right mandible was demonstrated, along with enlarged lymph nodes at both sides of the neck [Figure 1]. As a part of the staging and planning procedures a laryngoscopy and a pharyngoscopy were performed, as well as biopsies and marking of the boundaries of the tumor with a safety distance. Upon histopathological confirmation of the suspected OSCC diagnosis, the case was discussed during a meeting with our Multidisciplinary Team (MDT), when a primary surgical treatment was recommended. In March 2013 a tumor resection combined with a right mandibulectomy, a neck dissection at level I-V at the right side and level I-III at the left side and a simultaneous reconstruction with a vascular fibular transplant were performed. The histopathological examination of the resected specimen showed a moderately differentiated squamous cell carcinoma with perineural infiltration and one nodal metastasis (pT3 pN1 (1/46) L0 V2 Pn1 G3, local R0) [Figure 2a]. The histology of a macroscopically visible nodule in the neck dissection specimen showed a hemangiosis carcinomatosa (V2) [Figure 2b]. During his stay a heparin-induced thrombocytopenia (HIT) was diagnosed. After a consultation with our Department of Transfusion Medicine the thromboprophylaxis regime was switched from Enoxaparin 20 mg s.c. 1-0-0 to Danaparoid 750 IE s.c. 1-0-1. The postoperative MDT recommended radiation therapy. A locoregional radiation was performed from April 2013 to June 2013 (intensity modulated radiation therapy (IMRT) with a daily fraction of 2,1/1,8 GyHD 65,1/55,8 Gy). After radiation the patient joined our six-weekly tumor-follow-up at our department. Seven months later the decision for the removal of the osteosynthesis plate (Stryker GmbH & Co.KG, Duisburg, Germany, 2.7 mm) of the lower jaw was made and a panoramic radiograph as well as a CT were performed [Figure 3]. It showed no signs for a relapse of the OSCC but pseudarthrosis at two sides of the reconstructed mandible. The pseudarthrosis was treated with a bone graft from the iliac crest. During the surgical procedure several soft tissue biopsies from the right floor of the mouth were taken. No relapse was found within the obtained material. Five months later the decision for the removal of the miniplates (Stryker GmbH & Co.KG, Duisburg, Germany, 2.0 mm), used for the fixation of the iliac crest bone graft, was made. In March 2014 the patient showed up for surgery [Figure 4]. During the clinical examination the patient reported that he has detected a growing mass at his left upper arm about four weeks ago. Clinically the tumor was miming an abscess with redness, heat, swelling and moderate pain. The upper arm was x-rayed and a swelling of the soft-tissue in projection to the lower margin of the deltoid muscle was found [Figure 5]. Sonographically a differentiation between abscess and soft-tissue tumor could not be done [Figure 6]. Therefore MR-Imaging was performed. The MRI described an inhomogeneous tumor at the proximal upper arm with circular extent around the humerus up to the axilla [Figure 7]. Local arrosions of the corticalis suggested malignancy; however, a differentiation between a soft-tissue tumor and a metastatic tumor spread was not possible. Therefore a biopsy was taken and the histological examination revealed a widely necrotic moderately differentiated keratinizing squamous cell carcinoma (G2) [Figure 8]. The case was discussed in an Interdisciplinary MDT. Due to the tumor localization - wrapping the humerus and in close proximity to the brachial plexus and the vascular bundle - the decision to manage it with radiation was made. The patient decided to start radiation therapy at his local hospital. When he showed up for therapy a picture was taken. Noticeable was the rapid progress of the metastasis after the biopsy was taken with a clear infiltration of the skin. Experimental and clinical studies have shown rapid local growth after traumatic interventions, possibly explaining the progress [Figure 9] [4-9].

Distant metastases from OSCCs occur at many sites with an incidence of 5 - 25% [10]. The most common sites of metastasis are the (regional) lymph nodes, followed by lung metastasis [2]. Skin metastasis can occur in diverse head and neck tumor entities as well as in the OSCC of the floor of the mouth but are less common [11]. Rare cases of soft tissue metastases in head and neck SCC [12] as well as bone marrow carcinosis have been reported [13]. These events usually occurred several years after the primary tumor diagnosis but a distant soft tissue metastasis of the upper arm of an OSCC of the floor of the mouth has never been described until now [14]. Shingaki et al. reported that distant metastasis usually occur in patients with regional lymph node metastasis [3]. In our patient a perineural invasion [Figure 2a] as well as a hemangiosis carcinomatosa (V2) were described [Figure 2b]. Several studies have reported a significant association between lymph node metastasis and perineural invasion as well as a higher probability of distant metastasis accompanied with a reduced five-year survival rate [15-17]. Furthermore Matos and colleagues showed a statistically significant higher frequency of metastasis of patients with a perivascular invasion in SCC of the tongue [18-20]. But there is still no sufficient evidence of histopathological risk factors developing distant (soft tissue) metastasis for OSCC of the floor of the mouth as this seems to be a rare event. Although metastases to the soft tissue generally don’t seem to be an infrequent finding, they are rare in comparison to metastases to parenchymal organs, despite the fact that soft tissue comprises about 55% of human body mass [14]. Based on the facts of this finding specific local conditions at this sites (for example changes in pH and metabolite accumulation, local temperature, characteristics of blood flow) have been suggested [21-23] According to the literature adenocarcinoma is the most common histologic tumor entity that gives distant soft tissue metastases, followed by renal cell carcinoma, squamous cell carcinoma and melanoma [14,24-26].

The macroscopic appearance of the metastatic site led to the differential diagnosis of an abscess in the first place. The typical signs of inflammation were present and the fast growing mass also made an abscess quite likely. Due to the fact that the patient had a tumor history and that general symptoms usually caused by an abscess of that size were missing taking a closer look was obligatory.

The humerus x-ray was not adjuvant making differential diagnosis. Abscess, metastasis and soft tissue tumors could not be distinguished. Therefore a sonography was performed. A typical abscess formation, such as a cavity formation could not be demonstrated, thus a MRI was performed. The MRI was able to illustrate the size and dignity of the mass but a diagnosis still could not be confirmed.

Therefore a biopsy became mandatory. The biopsy revealed infiltration by a moderately differentiated squamous cell carcinoma (G2) matching a metastasis from the known OSCC of the floor of the mouth.

Even with advanced diagnostic tools the radiological appearance was unclear. Conventional methods did not contribute to the diagnosis. The only advantage of the MRI was in accurately describing the dimensions of the tumor. It is a well-known issue that a radiological discrimination of a lesion’s dignity may be limited [27-29]. The most important differential diagnostic discrimination needs to be made between metastastic carcinomas and primary soft tissue sarcoma, since it has serious prognostic and therapeutic implications.

In the presented case the close tumor-follow-up enabled the detection of the metastasis after four weeks of growing. Obviously it would have been even better if the patient had shown up immediately after the first symptoms appeared. This illustrates the necessity for a detailed instruction of the patient at the beginning of the tumor-follow-up care; patients need to be encouraged to present and report each symptom that are uncommon, so that a diagnosis can be made in time. The quality of the follow-up care is thus heavily depended on the patient’s compliance.

Another problem comes with the CT protocol. The usual CT protocol within a tumor-follow-up excludes the arms during thorax or head and neck protocols to lower the radiation dose. The last CT was performed in November 2013. It remains unclear if a metastatic lesion may have been diagnosed to that point. Even the routine CT performed every year during tumor-follow-up would not have been able to illustrate the metastasis using the routine protocols.

## Conclusions

During a tumor-follow-up the clinical examination should also include rare metastasis sites. The physician should take time for detailed history taking as well as a thorough clinical examination. The physician should illustrate to the patient the importance of early visits if unusual physically changes like swellings occur.

The differential diagnosis of metastasis is pivotal to deliver the most appropriate treatment and should always be systematically considered in a patient with tumor history.

Due to the fact that patients with regional lymph node metastases have a higher probability to develop distant metastasis [3] a more detailed screening might be considered – especially when hemangiosis carcinomatosa was found histologically or macroscopically. The follow-up computed tomography could include the arms for example. Nonetheless higher radiation dose should be traded off the probability of detecting distant metastasis.

### Consent

Written informed consent was obtained from the patient for publication of this case report and any accompanying images. A copy of the written consent is available for review by the editor of this journal.

## Abbreviations

OSCC: Oral squamous cell carcinoma
CT: Computed tomography
MDT: Multidisciplinary team
HIT: Heparin-induced thrombocytopenia
IMRT: Intensity modulated radiation therapy

## References

[1] Listl S, Jansen L, Stenzinger A, Freier K, Emrich K, Holleczek B (2013). Survival of patients with oral cavity cancer in Germany. PLoS One.

[2] Jones AS, Morar P, Phillips DE, Field JK, Husband D, Helliwell TR (1995). Second primary tumors in patients with head and neck squamous cell carcinoma. Cancer.

[3] Shingaki S, Suzuki I, Kobayashi T, Nakajima T (1996). Predicting factors for distant metastases in head and neck carcinomas: an analysis of 103 patients with locoregional control. J Oral Maxillofacial Surgery Off J Am Assoc Oral Maxillofacial Surgeons.

[4] Murthy SM, Goldschmidt RA, Rao LN, Ammirati M, Buchmann T, Scanlon EF (1989). The influence of surgical trauma on experimental metastasis. Cancer.

[5] Bogden AE, Moreau JP, Eden PA (1997). Proliferative response of human and animal tumours to surgical wounding of normal tissues: onset, duration and inhibition. Br J Cancer.

[6] Lange PH, Hekmat K, Bosl G, Kennedy BJ, Fraley EE (1980). Acclerated growth of testicular cancer after cytoreductive surgery. Cancer.

[7] Peeters CF, de Waal RM, Wobbes T, Westphal JR, Ruers TJ (2006). Outgrowth of human liver metastases after resection of the primary colorectal tumor: a shift in the balance between apoptosis and proliferation. Int J Cancer J Int Du Cancer.

[8] Takahashi H, Umeda M, Takahashi Y, Matsui T, Shigeta T, Minamikawa T (2013). Influence of preoperative dental procedures on the prognosis of patients with squamous cell carcinoma of the gingiva. British J Oral Maxillofacial Surgery.

[9] Ohtake K, Shingaki S, Nakajima T (1990). Effects of incision and irradiation on regional lymph node metastasis in carcinoma of the hamster tongue. Oral Surgery Oral Medicine Oral Pathol.

[10] Calhoun KH, Fulmer P, Weiss R, Hokanson JA (1994). Distant metastases from head and neck squamous cell carcinomas. Laryngoscope.

[11] Pitman KT, Johnson JT (1999). Skin metastases from head and neck squamous cell carcinoma: incidence and impact. Head Neck.

[12] Kulahci Y, Zor F, Onguru O, Bozkurt M, Duman H (2009). Distant muscular (rectus femoris) metastasis from laryngeal squamous cell carcinoma. J Laryngol Otol.

[13] Frolich K, Alzoubi A, Muller J, Kleinsasser N (2013). Bone marrow carcinosis in head and neck carcinoma in a young adult. J Oral Maxillofacial Surgery Off J Am Assoc Oral Maxillofacial Surgeons.

[14] Plaza JA, Perez-Montiel D, Mayerson J, Morrison C, Suster S (2008). Metastases to soft tissue: a review of 118 cases over a 30-year period. Cancer.

[15] Chen YW, Yu EH, Wu TH, Lo WL, Li WY, Kao SY (2008). Histopathological factors affecting nodal metastasis in tongue cancer: analysis of 94 patients in Taiwan. Int J Oral Maxillofac Surg.

[16] Sparano A, Weinstein G, Chalian A, Yodul M, Weber R (2004). Multivariate predictors of occult neck metastasis in early oral tongue cancer. Otolaryngol Head Neck Surgery Off J Am Academy Otolaryngol Head Neck Surgery.

[17] Rahima B, Shingaki S, Nagata M, Saito C (2004). Prognostic significance of perineural invasion in oral and oropharyngeal carcinoma. Oral Surgery Oral Med Oral Pathol Oral Radiol Endodontics.

[18] de Matos FR, Lima E, Queiroz LM, da Silveira EJ (2012). Analysis of inflammatory infiltrate, perineural invasion, and risk score can indicate concurrent metastasis in squamous cell carcinoma of the tongue. J Oral Maxillofacial Surgery Off J Am Assoc Oral Maxillofacial Surgeons.

[19] Close LG, Burns DK, Reisch J, Schaefer SD (1987). Microvascular invasion in cancer of the oral cavity and oropharynx. Archives Otolaryngol Head Neck Surgery.

[20] Jones HB, Sykes A, Bayman N, Sloan P, Swindell R, Patel M (2009). The impact of lymphovascular invasion on survival in oral carcinoma. Oral Oncol.

[21] Herring CL, Harrelson JM, Scully SP (1998). Metastatic carcinoma to skeletal muscle. A report of 15 patients. Clin Orthop Relat Res.

[22] Stulc JP, Petrelli NJ, Herrera L, Lopez CL, Mittelman A (1985). Isolated metachronous metastases to soft tissues of the buttock from a colonic adenocarcinoma. Dis Colon Rectum.

[23] Magee T, Rosenthal H (2002). Skeletal muscle metastases at sites of documented trauma. AJR Am J Roentgenol.

[24] Bibi C, Benmeir P, Maor E, Sagi A (1993). Hand metastasis from renal cell carcinoma with no bone involvement. Ann Plast Surg.

[25] Alburquerque TL, Ortin A, Cacho J (1987). Metastasis in deep calf muscles as first manifestation of bronchus adenocarcinoma. Am J Med.

[26] Amano Y, Kumazaki T (1996). Gastric carcinoma metastasis to calf muscles: MR findings. Radiat Med.

[27] Curtin HD, Ishwaran H, Mancuso AA, Dalley RW, Caudry DJ, McNeil BJ (1998). Comparison of CT and MR imaging in staging of neck metastases. Radiology.

[28] Koc O, Paksoy Y, Erayman I, Kivrak AS, Arbag H (2007). Role of diffusion weighted MR in the discrimination diagnosis of the cystic and/or necrotic head and neck lesions. Eur J Radiol.

[29] Wang J, Takashima S, Takayama F, Kawakami S, Saito A, Matsushita T (2001). Head and neck lesions: characterization with diffusion-weighted echo-planar MR imaging. Radiology.

